# Machine learning integrates region-specific microbial signatures to distinguish geographically adjacent populations within a province

**DOI:** 10.3389/fmicb.2025.1586195

**Published:** 2025-07-11

**Authors:** Li Luo, Bangwei Chen, Shengyin Zeng, Yaxin Li, Xiaolin Chen, Jianguo Zhang, Xiangjie Guo, Shujin Li, Lei Ruan, Shida Zhu, Cairong Gao, Cuntai Zhang, Tao Li

**Affiliations:** ^1^Department of Pathology, School of Forensic Medicine, Shanxi Medical University, Taiyuan, China; ^2^BGI Genomics, BGI-Shenzhen, Shenzhen, China; ^3^School of Biology and Biological Engineering, South China University of Technology, Guangzhou, China; ^4^College of Life Sciences, University of Chinese Academy of Sciences, Beijing, China; ^5^School of Life Sciences, South China Normal University, Guangzhou, China; ^6^Hebei Key Laboratory of Forensic Medicine, Research Unit of Digestive Tract Microecosystem Pharmacology and Toxicology, College of Forensic Medicine, Chinese Academy of Medical Sciences, Hebei Medical University, Shijiazhuang, China; ^7^Department of Geriatrics, Tongji Hospital, Tongji Medical College, Huazhong University of Science and Technology, Wuhan, China

**Keywords:** intestinal bacteria, machine learning, geographic locations, metagenomics, forensic microbiology

## Abstract

**Background:**

The individual specificity and temporal stability of the human gut microbiota have revealed significant compositional differences across geographical provenances. However, the gut microbiota variations among people residing in different regions within a province remain enigmatic.

**Methods:**

Shotgun metagenomics sequencing was performed to analyze the gut microbiota of 381 unrelated Chinese Han individuals living in two cities (Wuhan and Shiyan) of Hubei Province. To obtain the optimal model that can distinguish geographically close populations, three machine learning (ML) algorithms based on microbiota or functions were employed.

**Results:**

Significant differences in microbial *α* diversity and *β* diversity were observed. *Flavonifractor plautii* and *Bacteroides stercoris* were region-specific markers that presented higher relative abundances in Wuhan individuals. By utilizing the genus-level index commonly used for 16 s RNA as the base model, the prediction accuracy was greatly improved when species and functional data were added. Among the three ML algorithms, the random forest algorithm achieved the best performance, with an AUC of 0.943.

**Conclusion:**

The gut microbiota of individuals residing in the same province is significantly similar; however, pronounced differences in bacterial composition were noted between individuals. Integrating the gut microbiota and functions using machine learning algorithm can distinguish people from geographically close environments, offering a foundation for determining geographical origin through the gut microbiota. Moreover, a deeper understanding of host-specific associations may offer valuable forensic and clinical assistance.

## Introduction

The human gut harbors complex microbial ecosystems with individual specificity and temporal stability that has an important impact on human health (Kuziel and Rakoff-Nahoum, 2022). Host factors, including geography, sex, body mass index (BMI), and age, play essential roles in shaping the composition and inter-individual variance of the gut microbiota (Dong et al., 2023; Wang et al., 2023; Pang et al., 2023). Early in 2012, Yatsunenko et al. (2012) emphasized that extensive sampling of diverse healthy individuals across varying geographic locations could discover their unique gut microbiota. Nowadays, numerous studies have been evaluated microbiota ability across continents, countries and ethnicities, revealing origin of the study participants significantly influences the observed differences in the microbiota (Handsley-Davis et al., 2022; Ogai et al., 2022; Cho and Eom, 2021). Cheng et al. (2021) employed amplicon sequencing on different continents and observed the effects of geographical location on microbiota profiles from China and Spain. Zhang et al. (2024) constructed a province-prediction model based on 3,224 individuals and demonstrated that microbiota compositions in host’s geographical location were affected by personal eating habits. Therefore, we inferred whether there are differences in gut microbiota among people living in different regions of the same province.

Machine learning (ML) represents a versatile suite of tools for discerning patterns and relationships in complex data, thereby playing a vital role in microbiology (Asnicar et al., 2024). Due to the high-dimensional and sparse characteristics of microbial data, ML can fit complex multi-dimensional interactions to achieve quantitative analysis and accurate prediction (Asnicar et al., 2024; Hernandez Medina et al., 2022). The combination of high-dimensional microbial data and ML offers distinct advantages in terms of temporal stability, geographic specificity, and automatic prediction (Xu et al., 2023), which can provide a more accurate decision-making basis for the application of microbiota in forensic practice (Yuan et al., 2023). To date, forensic scientists have been conducted to predict host characteristics by analyzing microbial profiles via ML, such as random forest (RF) (Liang et al., 2023), linear discriminant analysis (LDA) (Wang et al., 2022), support vector machine (SVM), logistic regression (LR) (Li et al., 2023), and so on. Yao et al. (2021) performed RF analysis to build a microbiota-based province-prediction model and realized the geographical tracing of unknown samples in Henan, Guangdong, and Xinjiang populations. Additionally, they further studied the characteristics of the microbial community of individuals living in three regions of Guangdong province and obtained an overall accuracy of 0.759 using RF (Huang et al., 2022). A meta-analysis of more than 3,000 people from 17 countries demonstrated that the feature-based ML model succeeded in the same country classification but had limited transferability to others (Chanda and De, 2024). Another study investigated the differences in the human microbiota across four distinct regions in China and explored the potential of RF to predict an individual’s geographical origin based on their microbiome data (Lei et al., 2025). However, the generalized ability of previous findings in ML models based on the microbiota has been limited by amplicon sequencing and variations among continents or provinces within countries.

Here, shotgun metagenomics sequencing was used to identify the gut microbiota profiles of 381 volunteers from two cities in Hubei Province, China (Supplementary Figure S1). Additionally, our study established an optimal prediction model to distinguish between participants from different regions. Moreover, we confirmed that integrating the gut microbiota and functions might be able to distinguish geographically adjacent populations within a province.

## Materials and methods

### Cohort description and sample collection

A total of 381 healthy individuals of Han nationality, originating from the Hubei Province, China, were enrolled. The inclusion criteria were as follows: (1) were >18 years old; (2) had no cancer, cardiovascular, or intestinal-related diseases; and (3) had no record of antibiotic usage in the previous 3 months. Stool samples were gathered and promptly stored at −80°C until DNA extraction. Moreover, blood and urine samples were analyzed by clinicians to acquire biochemical parameters. Demographic information (sex, age, height, weight, region) and lifestyle information (smoking) were collected via a questionnaire. Additionally, BMI = weight (kg)/height^2^ (m^2^). This study was performed in accordance with the Declaration of Helsinki and approved by the Ethics Committee of Tongji Medical College, Huazhong University of Science and Technology (2020S146). All the participants provided written informed consent.

### Metagenomics sequencing of stool samples

DNA was extracted from the stool samples using the MGIEasy Kit (MGI, Shenzhen, China). Approximately 500 ng of isolated DNA was used for library preparation and 100-bp single-end reads were sequenced on the DNBSEQ-T10 platform (MGI, Shenzhen, China). Low-quality reads were removed using SOAPnuke v2.1.7 (Chen et al., 2018). Contaminating human reads were filtered using Bowtie2 v2.5.0 and gcc v10.4.0 (reference database: GRCh38) with default parameters (Langmead and Salzberg, 2012). Taxonomic profiling of the bacterial community was performed using MetaPhlan v3.0.13 (Beghini et al., 2021). The relative abundances of each phylum, genus, and species were determined by aggregating the relative abundance of their annotated genes per individual. Rarefaction and extrapolation (R/E) sampling curves for estimation of total richness of microbial features in the population were constructed using a sample size-based interpolation/extrapolation algorithm implemented in the iNEXT package for R (Hsieh et al., 2016).

### Functional profiling of the gut microbiota

The microbial metabolic pathways were conducted with HUMAnN v3.1.1 (Beghini et al., 2021) for profiling the abundance of microbial metabolic pathways. After filtering the unmapped and unintegrated pathways, the remaining MetaCyc pathways underwent max-min normalization, and the relative abundances were recalculated. Furthermore, Spearman’s correlation between the gut microbiota and pathways was conducted. Only the interaction of absolute spearman rho greater than 0.3 and false discovery rate (*FDR*) less than 0.05 were screened. The co-occurrence network was visualized by Cytoscape v3.10.2 (Shannon et al., 2003). Additionally, the rewiring and community changes in the microbiota-pathway networks were quantified by NetShift (Kuntal et al., 2019). The drivers behind NetShift were obtained by introducing a neighbor shift score combined with quantification of node intermediation to transform node neighbors in the network.

### Microbiota and functional feature comparisons across different regions

The relative abundances of microbiota compositional data and MetaCyc pathways were used for downstream analyses. Alpha diversity (Shannon, Simpson, and richness) and beta diversity based on bray-curtis distance were computed. Differences between groups were plotted using principal coordinate analysis (PcoA), and differential clustering of microbial communities/functions was assessed using permutational multivariate analysis of variance (PERMANOVA) with the adonis function. Genus-level enterotype analysis was performed using bray-curtis distance and K-Nearest Neighbors clustering. The microbiota variation explained by personal characteristics and smoking status was evaluated with the envfit function. Additionally, a linear model was employed to assess the impacts of characteristics on the variance of each species after adjusting the smoking status. The network properties of microbial co-occurrence, including edge number, vertex number, and average degree, were assessed using the igraph package (Csárdi et al., 2024). Moreover, the relationship between microbiota and clinical measurements was performed. To assess the impact of distinct microbial communities/functions, a rigorous analysis was performed utilizing the linear discriminant analysis (LDA) Effect Size (LefSE) analysis (Gao et al., 2024).

### Machine learning analysis

Microbiota profiles and pathways were pre-filtered for more than 5% prevalence using MaAsLin2 v1.16.0 (Mallick et al., 2021). To obtain more specific microbiota profiles/pathways, the second round of screening was performed using Boruta v8.0.0. RF, support vector machine (SVM), and xgboost were applied. Participants were divided into training set (*N* = 306: 158 in Shiyan and 148 in Wuhan) and testing set (*N* = 75: 39 in Shiyan and 36 in Wuhan), with a ratio of 8:2. Fivefold cross-validation was repeated three times to construct a classifier model based on the training set. To evaluate the performance of the model, the area under the curve (AUC), accuracy, average precision (AP), and F1 score were calculated in the testing set. Besides, net reclassification improvement (NRI) and integrated discrimination improvement (IDI) were used to assess the incremental predictive performance of outcomes. Moreover, the optimal region-prediction model was obtained based on AUC. Finally, the AUC of the optimal model was calculated according to the sex subgroup in testing set.

### Statistical analyses

All the statistical analyses were performed using R v4.3.2. Wilcoxon rank sum test and chi-square test were conducted to compare continuous variables and categorical variables, respectively. Alpha diversity, beta diversity, adonis, envfit, and enterotype analysis were applied to the vegan package (Oksanen et al., 2024). Multiple comparisons were corrected using the false discovery rate (*FDR*) algorithm.

## Results

### Overview of populations

To ascertain whether the gut microbiota can distinguish between populations that are relatively proximate to each other, 381 volunteers (aged 25–75 years) residing in two cities (Wuhan and Shiyan) within the Hubei Province of China were recruited. No significant differences were observed in sex, age, height, weight, and BMI between the two groups (Table 1).

**Table 1:** Demographic characteristics of participants with available fecal samples.

Characteristics	All (*N* = 381)	Shiyan (*N* = 197)	Wuhan (*N* = 184)	*p*-value
Sex				0.638
Women	71 (18.6%)	39 (19.8%)	32 (17.4%)	
Men	310 (81.4%)	158 (80.2%)	152 (82.6%)	
Age	47.0 [37.0; 54.0]	48.0 [41.0; 52.0]	47.0 [35.0; 56.0]	0.912
Height	170 [166; 174]	169 [165; 173]	170 [166; 175]	0.064
Weight	71.7 [64.7; 77.6]	71.0 [64.0; 77.0]	72.4 [65.0; 77.7]	0.318
BMI	24.7 [22.9; 26.6]	24.7 [22.9; 26.6]	24.6 [23.0; 26.6]	0.899
SBP	123 [115; 134]	122 [115; 131]	124 [114; 136]	0.280
TC	4.61 [4.00; 5.16]	4.70 [4.16; 5.33]	4.46 [3.82; 5.07]	0.008
Smoking				<0.001
No	322 (84.5%)	181 (91.9%)	141 (76.6%)	
Yes	59 (15.5%)	16 (8.12%)	43 (23.4%)	
Enterotype				0.138
*Prevotella*	64 (16.80%)	39 (19.80%)	25 (13.6%)	
*Bacteroides*	317 (83.20%)	158 (80.20%)	159 (86.4%)	

### Landscape of the gut microbiota across populations

**Figure 1:**
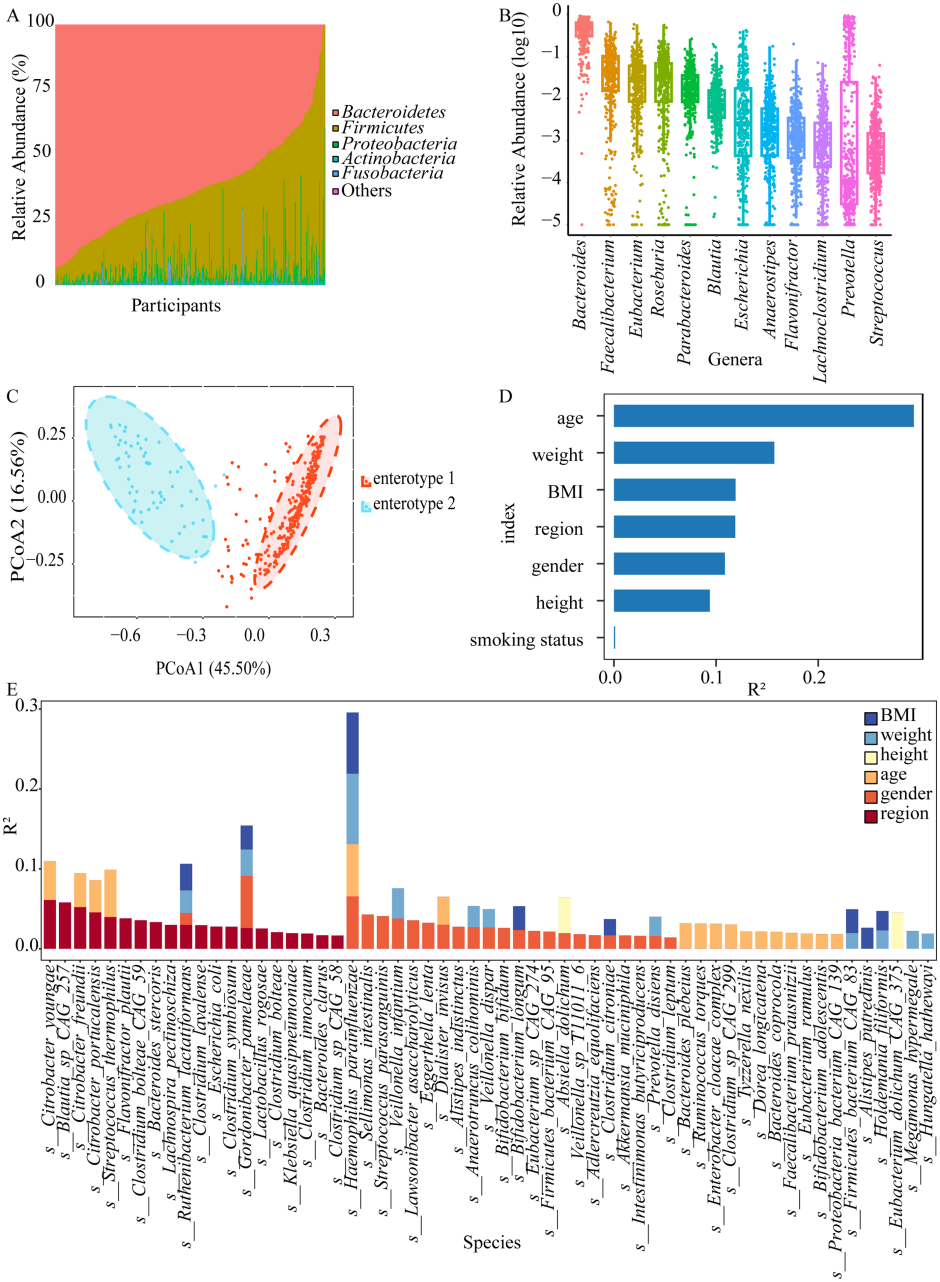
The gut microbiota composition of the Hubei population and associated personal characteristics. **(A)** Top six phyla by mean relative abundance for participants in Hubei. **(B)** The relative abundances (log10) of the core genera. **(C)** Two major enterotypes found in the stool samples from the Hubei population based on principal coordinate analysis (PcoA). **(D)** The effect sizes of personal characteristics associated with species variations were calculated with envfit (vegan), all characteristics with *FDR* < 0.05. **(E)** The bar plot displaying variations in each species explained by personal characteristics after adjusting smoking status, as estimated through the linear regression method (adjusted *R*^2^ > 1%, *FDR* < 0.05).

A total of 13 phyla, 218 genera, and 649 species were obtained by shotgun metagenomics sequencing (Supplementary Table S1). Our sample size allowed us to encompass over 80% of the total expected microbial features, as estimated by bootstrap analysis (Supplementary Figure S2A). The presence rates of these microbial features become relatively stable (within 80% of the numbers observed for the whole cohort) when at least 44% of the cohort is sampled (approximately 169 samples) through subsampling. However, the number of observed species increased with sample size, reaching an estimated total of 777 species at 2,000 samples, which indicated that other rare microbial species remained undiscovered. *Bacteroidetes*, *Firmicutes*, *Proteobatcteria*, *Actinobacteria*, and *Fusobacteria* were the five most abundant bacterial phyla in all the samples (Figure 1A). Gut microbiota composition exhibited significant variations among the populations, with the relative abundance of *Bacteroidetes* ranging from 0.08% to over 94.01%, for instance. To gain insight into the microbiota composition that may be potentially critical for the stability and consistency of the gut ecosystem, genera or species present in more than 90% of individuals (named “core microbiota”) were investigated. Twelve core genera (*Bacteroides*, *Prevotella*, *Parabacteroides*, *Streptococcus*, *Eubacterium*, *Anaerostipes*, *Blautia*, *Lachnoclostridium*, *Roseburia*, *Faecalibacterium*, *Flavonifractor*, and *Escherichia*) and ten core species (*Bacteroides ovatus*, *Bacteroides thetaiotaomicron*, *Bacteroides uniformis*, *Bacteroides vulgatus*, *Parabacteroides distasonis*, *Blautia wexlerae*, *Clostridium bolteae*, *Faecalibacterium prausnitzii*, *Flavonifractor plautii*, and *Escherichia coli*) were observed (Figure 1B; Supplementary Figure S2B). Interestingly, 90% of the core species were short-chain fatty acid (SCFA)-producing bacteria, with the exception of *Blautia wexlerae*. Furthermore, two distinct optimal enterotype clusters were identified (Figure 1C). Enterotype 1 (driven by *Bacteroides*) and enterotype 2 (*Prevotella*) accounted for 83.20 and 16.80% of the participants, with no difference between regions (Supplementary Figure S2C; Table 1). To identify the region and other characteristics related to the gut microbiota, seven variables based on species were evaluated by envfit. Age explained the largest variance (*R*^2^ = 0.294), followed by weight (*R*^2^ = 0.157), BMI (*R*^2^ = 0.119), and region (*R*^2^ = 0.119) (Figure 1D, *FDR* < 0.05). Moreover, the interpretation of individual characteristics by gut microbiota at the genus level showed that the region maintained the leading position (*R*^2^ = 0.121, *FDR* < 0.05, Supplementary Figure S2D). Interestingly, smoking had no explanatory power for species (*R*^2^ = 0.001, *FDR* = 0.662) and genus (*R*^2^ < 0.001, *FDR* = 0.879). Additionally, the variation attributed to personal characteristics for each species was estimated after adjusting the smoking status, and the primary determinant was subsequently identified. Fifty-nine species with cumulative adjusted *R*^2^ > 1% could be explained. Both sex and region dominantly accounted for the variations observed among species, region was the unique factor responsible for the inter-individual variations across 14 species, including *Flavonifractor plautii*, *Bacteroides stercoris*, *Blautia_sp_CAG_257*, 6 species from the *Clostridium* genus, and so on (Figure 1E; Supplementary Table S2). Hence, we speculated that region was an important factor contributing to variation in the gut microbiota.

**Figure 2:**
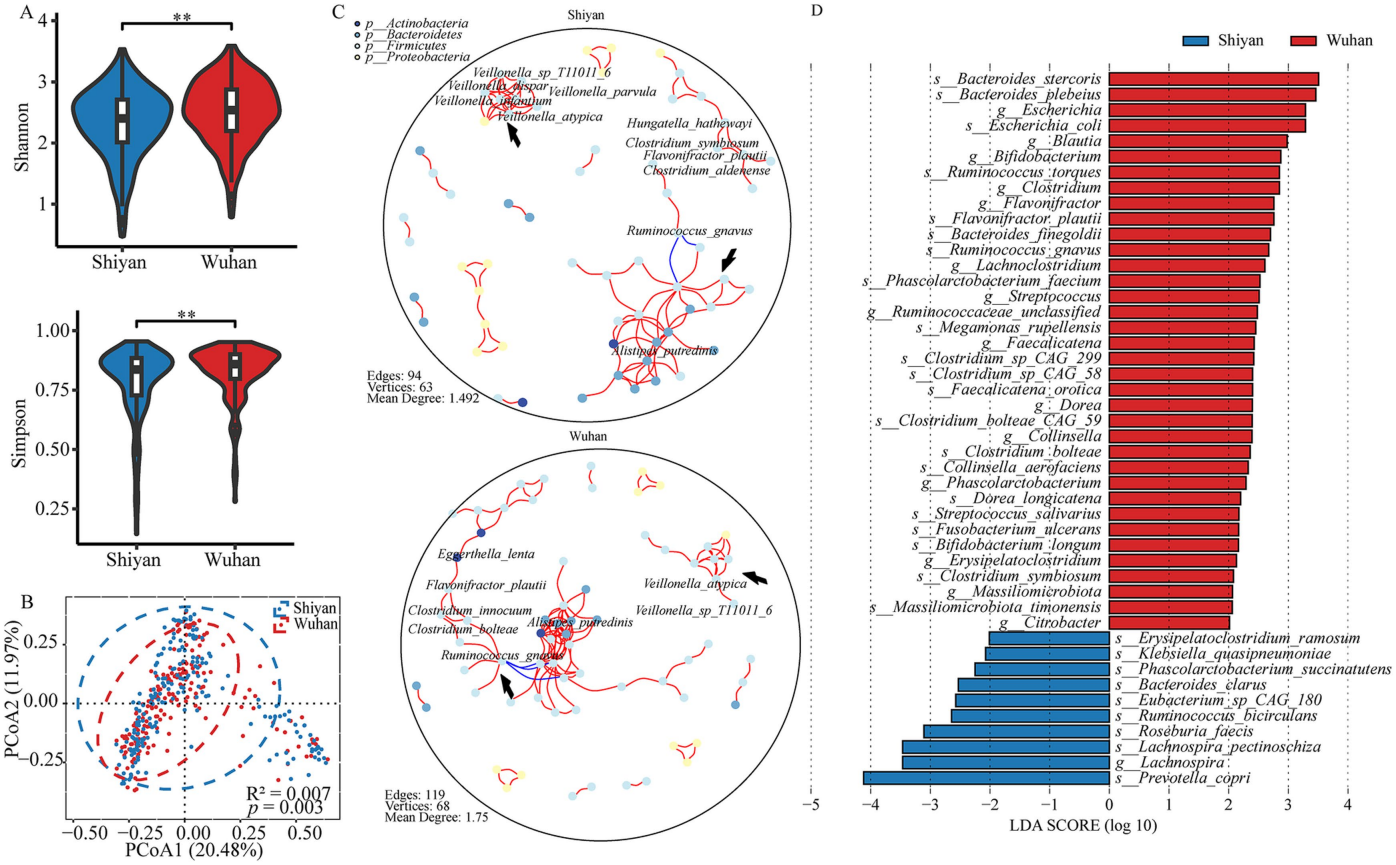
Differences in gut microbial community profiles between populations from different cities. **(A)** Alpha-diversities of microbial communities between Wuhan and Shiyan (*p* value was calculated using a wilcoxon test; **p < 0.01). **(B)** PcoA of pairwise bray–curtis distance showed the separated microbial composition between two populations (Adnois *R*^2^ = 0.007, *p* < 0.01). **(C)** The microbial interaction networks across 2 population, *FD*R < 0.05, cor > ±0.5. The arrows pointed to two sub-networks (*Veillonella* and *Alistipes putredinis*). **(D)** The bar graph of LDA scores showed the taxa with statistics difference between two groups. The LDA threshold was 2.

To further investigate the microbial structures of populations in geographical proximity, *α* diversity (Supplementary Table S3) and *β* diversity (Supplementary Table S4) were compared. As shown by the α diversity (Shannon, Simpson, and Richness) and β diversity, significant differences were observed (*p* < 0.05; Figures 2A,B; Supplementary Figure S3A). Next, the co-occurrence networks were performed (Figure 2C). The networks in both regions were fundamentally identical in structure, but they still had unique networks. Two sub-networks (A: *Veillonella*, B: *Alistipes putredinis*) differed across cities. For example, species from *Veillonella* displayed a close interaction in Shiyan, and *Ruminococcus gnavus* from sub-network B interacted with *Flavonifractor plautii* through *Clostridium* species in Wuhan. Additionally, core species *Flavonifractor plautii* was found in conjunction with *Hungatella hathewayi*, *Clostridium symbiosum*, and *Clostridium aldenense* in Shiyan, whereas it appeared with *Eggerthella lenta* and *Clostridium innocuum* in Wuhan. The LefSE result showed that 36 microbiota were enriched in Wuhan and 10 in Shiyan, which could explain the difference between the cities (Figure 2D, *FDR* < 0.05). Among these bacteria, 63% were species that determined the main dissimilarity. Wuhan group was characterized by *Bacteroides stercoris*, while *Prevotella copri* displayed a significant presence in Shiyan group. Additionally, we also found that *Flavonifractor plautii Ruminococcus gnavus*, *and Clostridium* species varied. Considering the imbalance and higher explanation of sex, the differential microbiota was identified (Supplementary Figure S3B). Eight microbiota co-existed by region and sex, including 4 species (*Ruminococcus bicirculans*, *Streptococcus salivarius*, *Bifidobacterium longum*, and *Clostridium symbiosum*) and 4 genera (*Lachnoclostridium*, *Streptococcus*, *Bifidobacterium*, and *Ruminococcaceae_unclassified*).

### Correlations between the gut microbiota and clinical parameters

**Figure 3:**
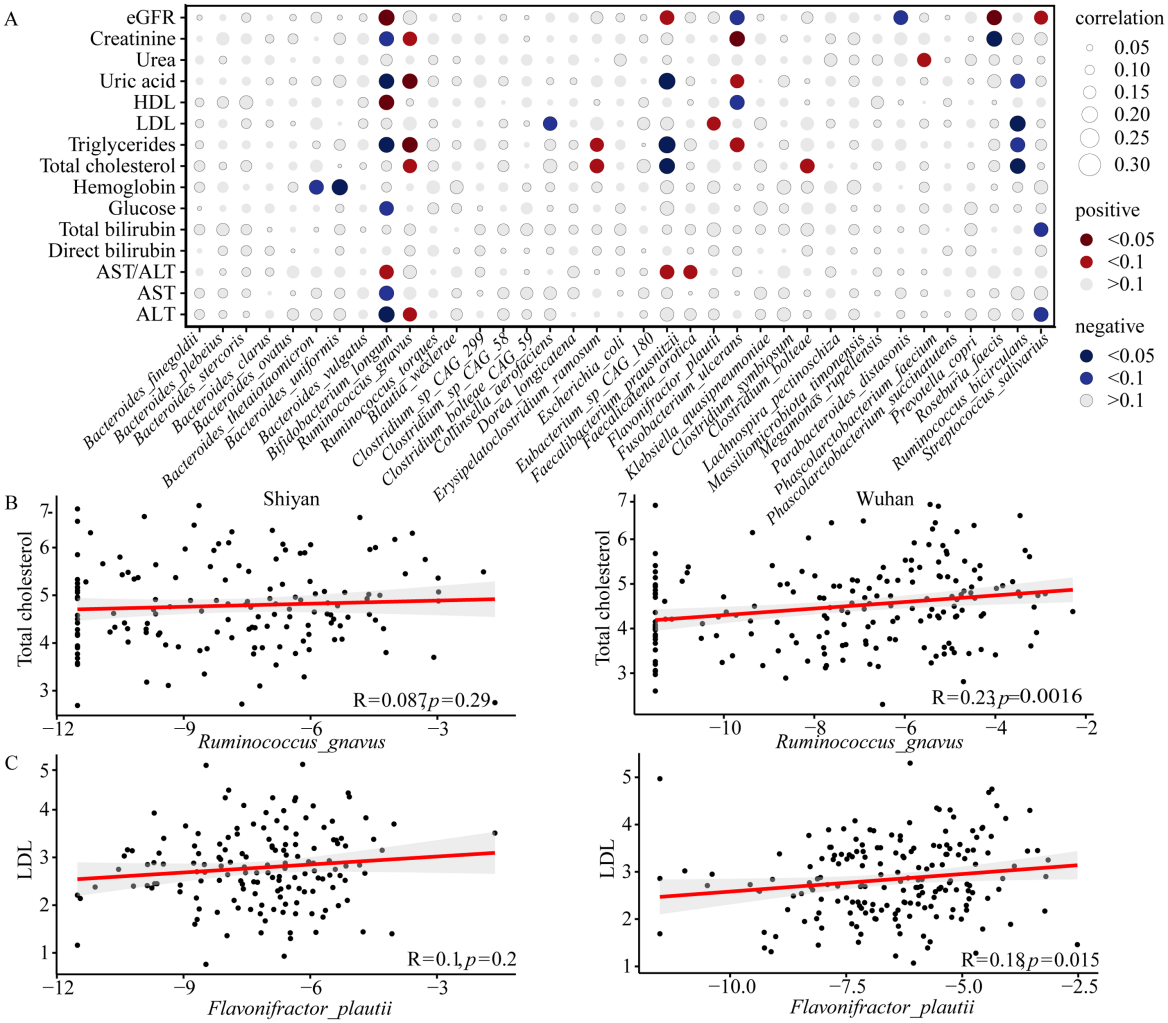
Correlation between gut species and clinical parameters. **(A)** Correlations between species abundance and clinical parameters were calculated through spearman correlation test with FDR correction. Only statistically significant correlations were shown where the correlation dot was color-intensified according to correlation direction (positive or negative) and coefficient size. **(B)** Correlations between the relative abundance (log) of *Ruminococcus gnavus* and total cholesterol in Wuhan and Shiyan. **(C)** Correlations between the relative abundance (log) of *Flavonifractor_plautii* and low-density lipoprotein (LDL) in Wuhan and Shiyan. Correlation coefficient Rho and statistical significance were calculated by spearman correlation analysis. eGFR, estimated glomerular filtration rate; HDL, high-density lipoprotein; LDL, low-density lipoprotein; AST, aspartate transaminase; ALT, alanine aminotransferase.

To gain insight into the potential functional associations between the gut microbiota and human health, the relationships between fifteen clinical parameters and 37 species (27 differential species, 7 core species, 3 both) were analyzed (Supplementary Table S5). A total of 15 bacteria exhibited a significant correlation with at least one clinical parameter (*FDR* < 0.1, Figure 3A). In all populations, *Bifidobacterium longum* had the most association with serum indicators of liver health, and *Ruminococcus bicirculans* showed negative correlation with serum lipid levels. Interestingly, *Bifidobacterium longum* and *Ruminococcus bicirculans* were negatively associated with alanine aminotransferase and total cholesterol only in their enriched populations, respectively (Supplementary Figure S4). Moreover, people with higher relative abundances of *Flavonifractor plautii* and *Ruminococcus gnavus* showed increases in low-density lipoprotein (LDL) or total cholesterol only in Wuhan (Figures 3B,C).

### Functionality variations of the gut microbiota

**Figure 4:**
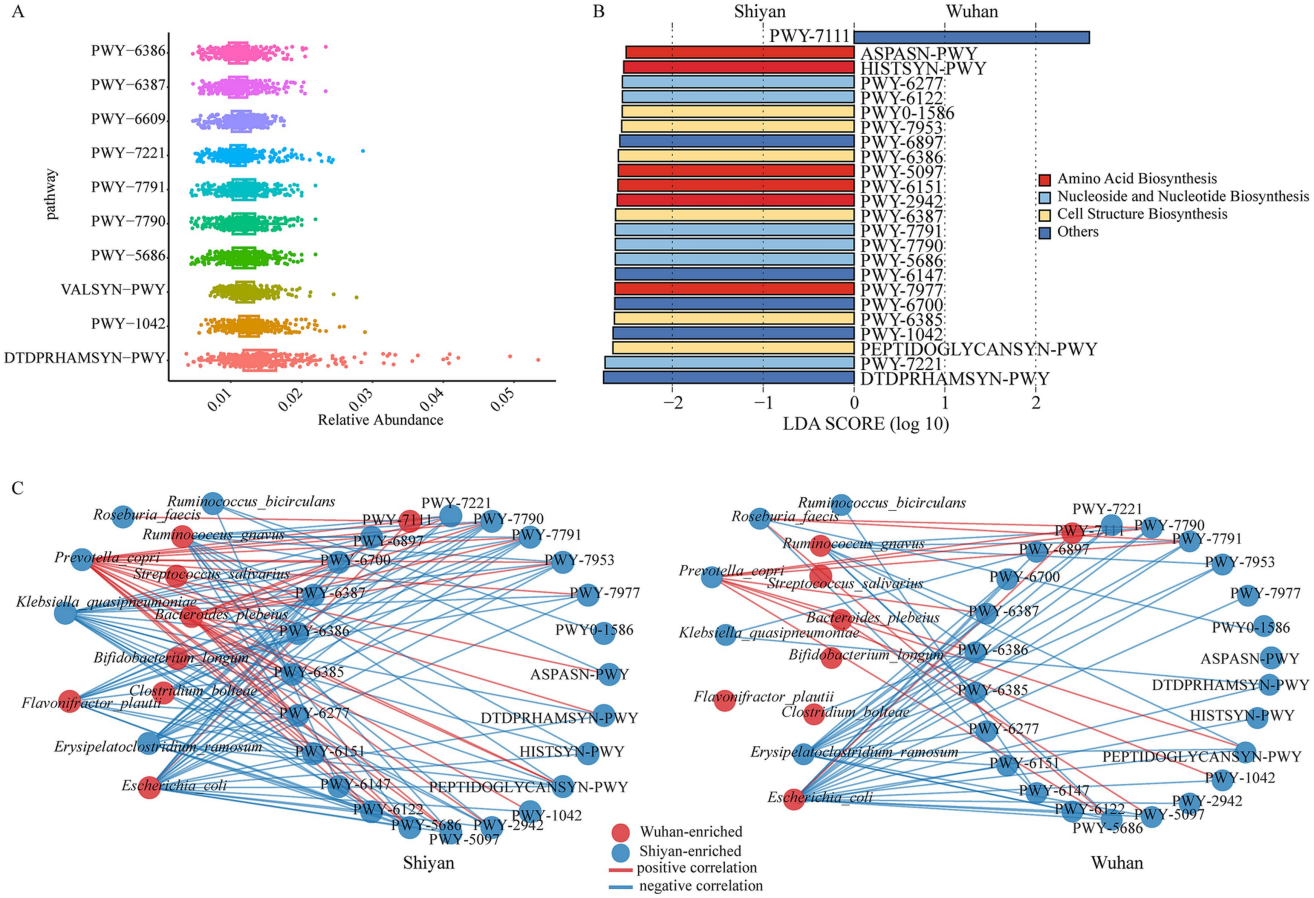
Microbial functional pathways altered in two populations. **(A)** The relative abundances of the core pathway. **(B)** The bar graph of LDA scores showed the pathway with statistics difference between two groups. The LDA threshold was 2.5. **(C)** Co-abundance network of differential species and pathways in two populations. Left, network in Shiyan individuals. Right, network in Wuhan individuals, arranged in the same order. Red circles, Wuhan-enriched; blue circles, Shiyan-enriched. Red edges, positive correlations; blue edges, negative correlations (*FD*R < 0.05, cor > ± 0.5).

To explore the metabolic pathways of gut microbiota that are potentially affected by regions, the relative abundances of MetaCyc pathways shared between individuals were calculated. A total of 515 pathways were found after filtering unmapped, unintegrated, and non-bacterial functions (Supplementary Table S6). The predominant metabolic pathway was the dTDP-*β*-L-rhamnose biosynthesis (DTDPRHAMSYN-PWY), which is the key process for the *in vivo* synthesis of deoxythymidine diphosphate L-rhamnose (dTDP-L-fucose) (Figure 4A). Above 52% of the pathways existed in more than 90% of individuals (named core pathways), and 174 pathways were observed in all people (Supplementary Table S6). According to their regions, 41 pathways appeared in only one. The overall structures based on their relative pathway abundances were significantly different between Wuhan and Shiyan (*p* < 0.05, Supplementary Figures S5A,B). Then, we explored the functional alterations between the regions. Differences in 24 pathways with LDA over 2.5 were found (Figure 4B), most of which were involved in amino acid biosynthesis, cell structure biosynthesis, as well as nucleoside and nucleotide biosynthesis. We found PWY-7111 (pyruvate fermentation to isobutanol) was the only abundant pathway in Wuhan. Subsequently, the bacterial composition of PWY-7111 was analyzed (Supplementary Table S7). Differential bacteria accounted for 15% (Shiyan) and 18% (Wuhan), and the relative abundance was different (Supplementary Figure S5C).

To further understand the relationships between differential bacteria and dissimilar functions across regions, two co-occurrence networks were constructed (Figure 4C; Supplementary Tables S8, S9). There were no links between the Wuhan-enriched bacteria (*Streptococcus salivarius*, *Bifidobacterium longum*, and *Flavonifractor plautii*) and pathways. In Shiyan, *Flavonifractor plautii* interacted with the biosynthesis of peptidoglycan, uridine monophosphate, and 5-aminoimidazole ribonucleotide. Meanwhile, another differential bacterium, *Ruminococcus gnavus*, was negatively associated with amino acid biosynthesis. An interesting finding was that both populations of *Ruminococcus gnavus* had pathways interacting with *Flavonifractor plautii*, which were specific to Shiyan. To quantify the changes observed among bacteria-pathway associations between Wuhan and Shiyan, network shift analyses were implemented (Supplementary Figure S6). When Wuhan was utilized as a control, *Klebsiella quasipneumoniae* and 5-aminoimidazole ribonucleotide biosynthesis metabolic pathways (PWY-6122 and PWY-6277) were particularly important drivers of Shiyan. Conversely, *Ruminococcus faecis* and vitamins biosynthesis (PWY-6897 and PWY-6147) were prominent drivers in Wuhan.

### Gut microbiota and functions differ geographically in proximity

**Figure 5:**
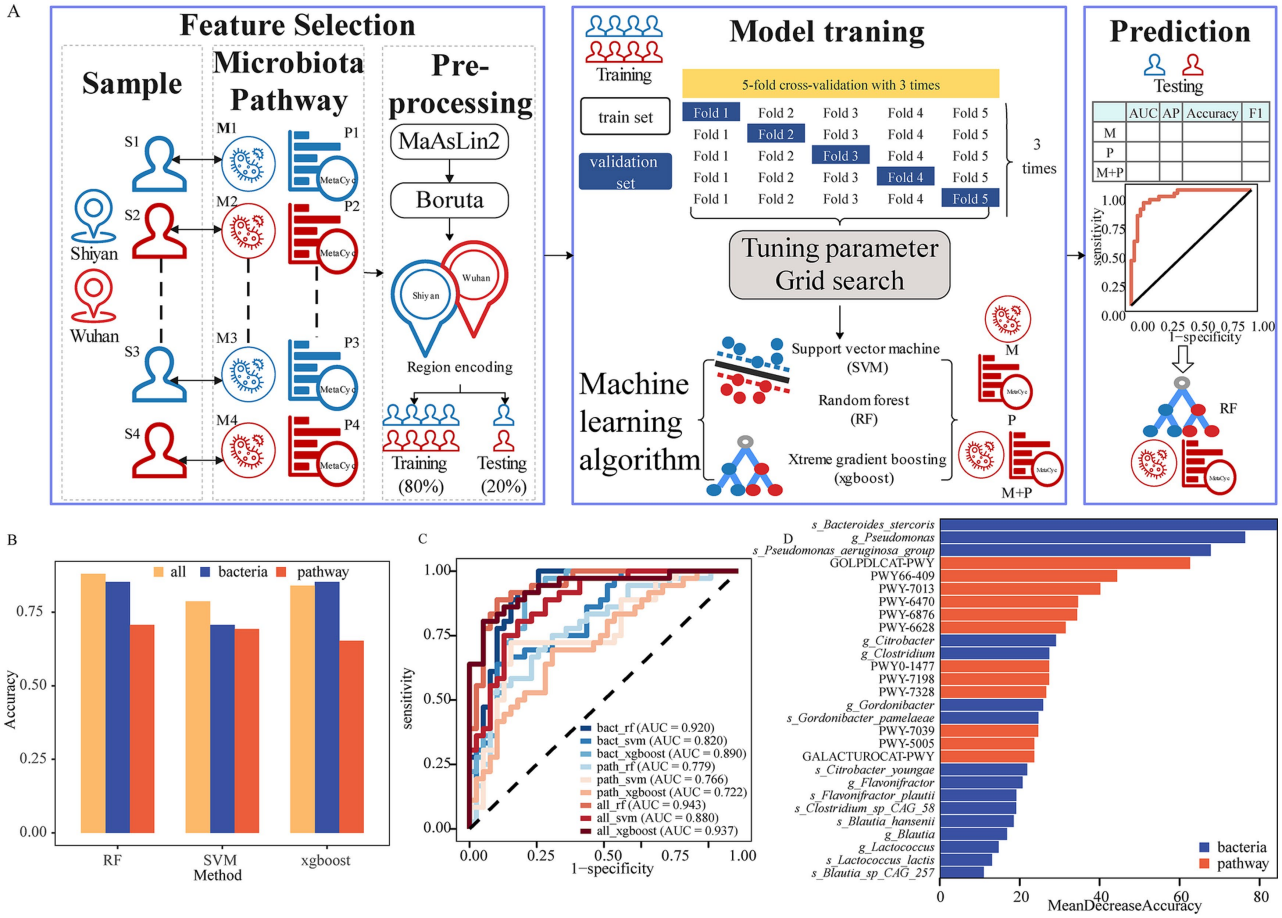
Gut microbiota differentiate geographically proximity. **(A)** Overview workflow for machine learning applications in our study. **(B)** The accuracy by data type and algorithm. **(C)** ROC curve of ML models based on bacteria and functions for predicting geographical proximity. **(D)** Twenty-eight important bacteria or functions to perform the prediction by random forest.

To rigorously determine the ML algorithm for predicting geographical proximity, three classical and widely utilized algorithms (RF, SVM, and xgboost) based on the composition and functionality of gut microbiota were implemented and compared (Figure 5A). We subsequently identified region-specific microbiota or pathways through two rounds of screening to avoid addressing dimensional disasters and overfitting, thereby ensuring the robustness and reliability of our predictions. Following the first filtering criteria of prevalence > 0.05% and *FDR* < 0.05 by MaAslin2, a total of 37 bacteria and 234 pathways were selected (Supplementary Table S10). The next step involved a refinement of the selected microbiota, adding species’ *α* diversity or pathways using a more stringent criterion of region specificity. The second round of screening was conducted by Boruta (*FDR* < 0.05). After applying this dual-pronged approach, 16 bacteria and 12 pathways were identified.

Based on the relative abundance of region-specific markers, 80% of the participants were divided into a training set and 20% into a testing set. The optimal parameters of each model for predicting the region were assessed by repeating the fivefold cross-validation three times on the training set. Model performance evaluation was conducted using AUC, average precision (AP), accuracy, and F1 score on the testing set (Supplementary Table S11). Our results indicated that the AUC of selected bacterial markers ranged from 0.820 to 0.920. Among the bacterial models, xgboost had the largest AP (0.780), RF and xgboost achieved identical accuracy (0.853), while RF exhibited the highest F1 score (0.864). For the pathway models, all the models performed poorly. Notably, the model integrating microbiota and pathways (integrated model) showed superior discriminative capabilities. Specifically, the accuracy of xgboost model did not improve, however, RF and SVM were the highest (Figure 5B). Nevertheless, other performances (AUC, AP, and F1 score) of integrated model were enhanced to a certain extent. Furthermore, we analyzed NRI and IDI to assess reclassification performance and improvement in discrimination of prediction model (Supplementary Table S12). The NRI and IDI had great improvements in the integrated model. These results suggested that the integrated model outperformed genus model.

Among the three algorithms, RF provided the best performance with the highest AUC of 0.943, followed by xgboost (AUC = 0.937) and SVM (AUC = 0.880) in the integrated model (Figure 5C). The accuracy, AP, and F1 score of RF were 0.880, 0.839, and 0.877, respectively (Supplementary Table S3). Finally, we determined which features of the gut microbiota and pathway were most important for the performance of the RF models. The important features included 9 species, 7 genera, and 12 functions (Figure 5D). *Bacteroides stercoris* was the strongest gut microbial marker for predicting the regions. Moreover, another 4 species (*Flavonifractor plautii*, *1 Clostridium* species, and 2 *Blautia* species) were observed to explain most of the region variations. Notably, six of the top 10 were pathways, indicating that function could play an important role in characterizing the region. Due to a high imbalance between sexes, we further analyzed the sex subgroups in the testing set. The AUC value was 0.943 (0.887–1) for males and 0.956 (0.851–1) for women (Supplementary Figure S7). These findings suggested that the overall model performed well across sexes.

## Discussion

We conducted a description of gut microbiota composition in Wuhan and Shiyan, Central China. To our knowledge, this study was the first effort utilizing shotgun sequencing in geographical proximity to characterize the gut microbiota of adults. *Bacteroidetes* and *Firmicutes* accounted for the majority of the microbial composition in healthy Hubei volunteers, which were consistent with the findings in Chinese participants (Lu et al., 2021; Ren et al., 2023). The enrichment of *Bacteroidetes* and *Firmicutes* corresponds to their involvement in carbohydrate metabolism and SCFA production (Ramos Meyers et al., 2022). Compared with the core genera and species of a healthy Chinese population, we found another 4 core species (*Bacteroides uniformis*, *Blautia wexlerae*, *Flavonifractor plautii*, and *Escherichia coli*) (Zhang et al., 2022). The difference could be attributed to variations in population, databases, and definitions. *Bacteroides* and *Prevotella* were the predominant enterotypes in our study, which fitted well with the enterotypes reported in Chinese populations (Syromyatnikov et al., 2022). Furthermore, region exhibited the strongest interactions with genera / species-level profiles. Although gut microbiota had been widely reported to vary across continents and countries, the robust association between geographic location and gut microbiota emphasized the obvious influence of geographical proximity on microbial composition in our study (Cheng et al., 2021; Lu et al., 2021).

Numerous studies underscore gut microbiota composition with sex (Koliada et al., 2021; Vriend et al., 2024), which suggested that regional differences might be influenced by the sex imbalance in this study. Meanwhile, several studies have found that changes in gut microbiota might have a significant impact on diseases in a sex-specific manner by regulating sex hormones, bile acids, lipids, and exogenous liver metabolism (Burra et al., 2024; Rosell-Diaz et al., 2024; Bardhan et al., 2024). *Bifidobacterium longum* was identified as one of the key species exhibiting sex-dependent variations in abundance. An animal study of chronic stress showed that *Bifidobacterium longum* was beneficial for water avoidance stress in rats, especially in females (Choi et al., 2024). *Streptococcus salivarius* was another sex difference species. Chen et al. (2020) suggested that individuals with relatively lower abundance of *Streptococcus salivarius* were more likely to have asthma. In our study, there were differences between these two species in terms of region and sex. Moreover, no links between these species and pathways were observed. Despite an imbalance in sex distribution, there was no difference in the distribution between the two regions. Considering that the explanatory power of sex was lower than that at the regional level, we speculated that the influence of sex on gut microbiota was relatively small in different regions.

Dietary intake can shape and modulate gut microbial composition and function across human populations, driving geographical differences (Parizadeh and Arrieta, 2023; Ross et al., 2024). Wuhan and Shiyan are located in two directions of Hubei Province, more than 500 km apart. Wuhan lies in east-central Hubei, a region rich in wetland resources where water products contribute to the local diet (Wikipedia, 2024). Shiyan is located in northwestern Hubei, bordering Henan, Chongqing, and Shaanxi Provinces, and its eating habits are easily influenced by these regions (Wikipedia, 2024). For this reason, we inferred that the distinctive characteristics of two cities were accompanied by dietary differences. Though *Bacteroides stercoris* was not the core species in healthy Chinese (Zhang et al., 2022), the RF model found it was the most important and was enriched in Wuhan, which might be important in distinguishing populations from the two regions. A cross-sectional study of forty-nine healthy volunteers showed that fiber-rich foods such as grain products and vegetables correlated positively with *Bacteroides stercoris* (Gaundal et al., *2022*). Moreover, Wuhan populations had less *Prevotella copri* potentially due to their high adherence to the Mediterranean diet (Wang et al., 2021), who might eat more fish and vegetables (Tsai et al., 2023). Additionally, *Ruminococcus faecis* was the driver of Wuhan, which was inversely associated with processed meat (Yu et al., 2021). Pyruvate fermentation to isobutanol (PWY-7111) was the only pathway enriched in Wuhan populations. Li et al. (2021) found that a higher healthy plant-based diet index score (fibers, plant proteins, whole grains, fruits, vegetables, nuts, and legumes) was associated with a greater relative abundance of pyruvate fermentation to isobutanol. Another study revealed that pyruvate fermentation to isobutanol was more common in herbivorous bats, suggesting that the microbiota might have adaptive functions to the plant-based diet (Ingala et al., 2021). Based on this, we suggested the residents of Wuhan prefer vegetables and fish to less processed meat.

As reported in a large cohort study with a wide geographic scale in China, food is a major mediating factor of geographic location on the gut microbiota (Zhang et al., 2024). Energy utilization during exercise promotes food breakdown and absorption by gut microbiota (Zhang et al., 2024). Lactate-utilizing species from *Veillonella*, which can improve physical performance constructed a strong interaction network in Shiyan populations, inferring participants might have a greater energy demand (Giacomini et al., 2023). Sleep quality and stress can also be impacted by diet and have been linked to gut microbiota composition (Kortman et al., 2024). Kortman et al. (2024) indicated that dairy-based products which improve sleep quality could decrease the relative abundance of *Flavonifractor plautii*. Chahwan et al. (2019) found a significant correlation between *Ruminococcus gnavus* and depression anxiety stress scale score. In our study, *Flavonifractor plautii* and *Ruminococcus gnavus* were increased in Wuhan populations, and the bacterial interactions were different. Our results speculated that differences in physical, sleep, and stress in the two populations might be associated with dietary diversity, which were closely related to geographical location. Alterations in the gut microbiota induced by differences in dietary habits may contribute to health status (Ross et al., 2024). A study from the Dutch Microbiome Project identified *Flavonifractor plautii* and *Ruminococcus gnavus* as signatures of disease (Gacesa et al., 2022). Another study revealed that *Flavonifractor plautii* and *Ruminococcus gnavus* had negative correlations with total and regional body fat (Wei et al., 2021). Due to no significant differences in weight and BMI between two groups, their possible effects on bacterial differences were excluded. Furthermore, *Flavonifractor plautii* and *Ruminococcus gnavus* were positively correlated with serum lipid levels only in Wuhan populations, implying the potential roles in lipid metabolism might differ between two populations. *Bifidobacterium longum* belongs to *Bifidobacterium* and is a common probiotic, which was another differential species between Shiyan and Wuhan. Zhao F *et al*. found that the gut *bifidobacterial* species in people from various geographic origins showed different responses to probiotic administration (Zhao et al., 2022). Moreover, *Bifidobacterium longum* was only correlated with ALT in the Wuhan populations. A randomized controlled trial demonstrated that *Bifidobacterium longum* does not affect liver dysfunction, but may treat liver dysfunction caused by medications in patients with depression (Gawlik-Kotelnicka et al., 2024). Furthermore, *Bifidobacterium longum* also showed significant differences between different sexes Therefore, regional and sex characteristics should be taken into consideration to ensure optimal therapeutic effects when using gut microbiota as probiotics. Additionally, 5-aminoimidazole ribonucleotide biosynthesis increased and were drivers of Shiyan people. Ma et al. (2021) showed that 5-aminoimidazole ribonucleotide biosynthesis were decreased in inflammatory bowel disease patients compared with healthy individuals. Hence, Wuhan participants might have poor sleep quality, high stress, and suboptimal health status. Geographical proximity differences in microbiota composition pointed to the underlying impact of dietary intake, lifestyle, and health status. Furthermore, personalized probiotic treatment based on individual microbiome profiles and geographical backgrounds was very important.

With the gradual improvement in human microbiota research, scholars found the differences mentioned above can be used to infer the geographic location information of individuals (Zhang et al., 2024; Ren et al., 2023). Machine learning analysis has a remarkable effect in tracing the geographical origin of unknown samples, and has great potential in scientific fields such as forensics, bacterial ecology, and other sciences (Walker and Datta, 2019). To determine whether the gut microbiota or pathways were able to distinguish between two geographically close populations, three ML algorithms (RF, xgboost, and SVM) were performed. RF can be trained and employed for prediction through multiple decision trees, which can effectively mitigate overfitting (Zhang et al., 2023). Xgboost contrasts RF as an efficient ensemble learning algorithm that improves prediction accuracy by sequentially building multiple decision trees in an attempt to reduce the errors of the preceding tree (Sun et al., 2025). SVM is a model that uses “support vectors” to construct the hyper-plane in a high-dimensional space (Maggioni and Spinelli, 2025). These algorithms were chosen for their robustness when working with high-dimensional data and small sample sizes, as well as their popularity and competitiveness in the microbiome field (Papoutsoglou et al., 2023; Ning et al., 2024). Feature selection, which is a common data preprocessing method in ML modeling, reduces model complexity and improves accuracy (Acikoglu and Tuncer, 2020). Finally, 16 bacteria and 12 pathways were identified by MaAslin2 and Boruta. Since 16 s rRNA amplicon sequencing could not be thoroughly analyzed at the species level, we used only the genus-level index as the base model. An important discovery was the substantial improvement in forecast precision when integrating both species and pathways index into the base model. This compelling outcome highlighted the influence of microbiota and their functions in enhancing predictive models for people in close geographical proximity. Among three ML algorithms, RF achieved the best performance, with an AUC of 0.943. Ryan (2019) also developed a RF classifier utilizing a dataset comprising 311 city microbiome samples and correctly classified 83.3% in city of origin for each sample. Feature importance scores of RF model indicated that prediction performance was not attributable to any single bacteria or metabolic pathway. Instead, it was the combination of both that played a pivotal role. Notably, the top three pathways were involved in alcohol degradation and purine nucleotide biosynthesis, which might differ between Wuhan and Shiyan populations, primarily owing to dietary variations. Additionally, the integrated model based on the RF performed well across sexes. Women showed slightly better discrimination ability, possibly due to fewer samples within the women subgroup. Consequently, both gut microbiota and function could reflect personal characteristics and their integration might predict the geographic origin of unknown individuals.

Although we have demonstrated that gut microbiota fingerprinting can be a potential tool for tracing population’s geographical origin (despite the population living in different cities from the same province), our study has several additional limitations. First, we chose only 2 cities from 1 province with a relatively small sample size that may or may not be representative of geographical proximity. Second, although the gut microbiota has been reported to be stable in the population, we cannot determine whether these important indicators are stable because we did not conduct longitudinal studies (Chen et al., 2018). Third, due to the lack of diet questionnaires and other lifestyle factors of the populations in the two cities, it is difficult to accurately assess the role of these factors in microbial changes. Finally, although the model demonstrated robustness across sexes, future studies should still consider differences between males and females to improve predictive accuracy.

Our study illustrates geographical factors accounted for a significant proportion of the variation in the gut microbiota. Although people from geographically close environments have similar microbiota profiles, they also have their own gut microbiota compositions. Integrating the gut microbiota and functions using machine learning algorithm can distinguish people from geographically close environments. In conclusion, it may be possible to determining geographical origin through the gut microbiota.

## Data Availability

Sequence data have been presented in this study have been deposited in the China National Center for Bioinformation / Beijing Institute of Genomics, Chinese Academy of Sciences with project number HRA009046. This data can be found here: https://ngdc.cncb.ac.cn/gsa-human/browse/HRA009046.
