# Supplementary material for: Machine learning integrates region-specific microbial signatures to distinguish geographically adjacent populations within a province
Source: Front Microbiol. 2025 Jul 11;16:1586195. doi: 10.3389/fmicb.2025.1586195 (PMC12289627; doi:10.3389/fmicb.2025.1586195)
Supplement: Supplementary file 1 [file Data_Sheet_1.docx]

**Supplementary Figures**


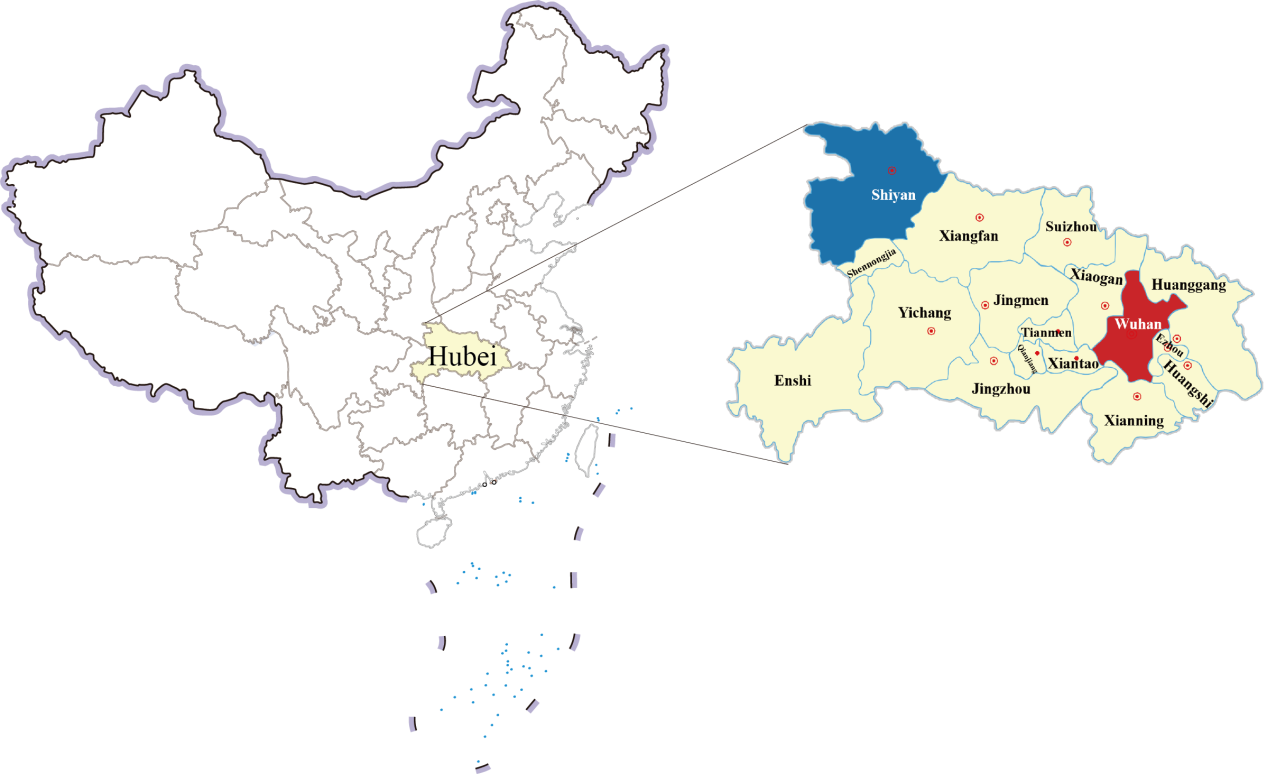


**Figure S1.** **Distribution map of Wuhan and Shiyan in Hubei Province.**


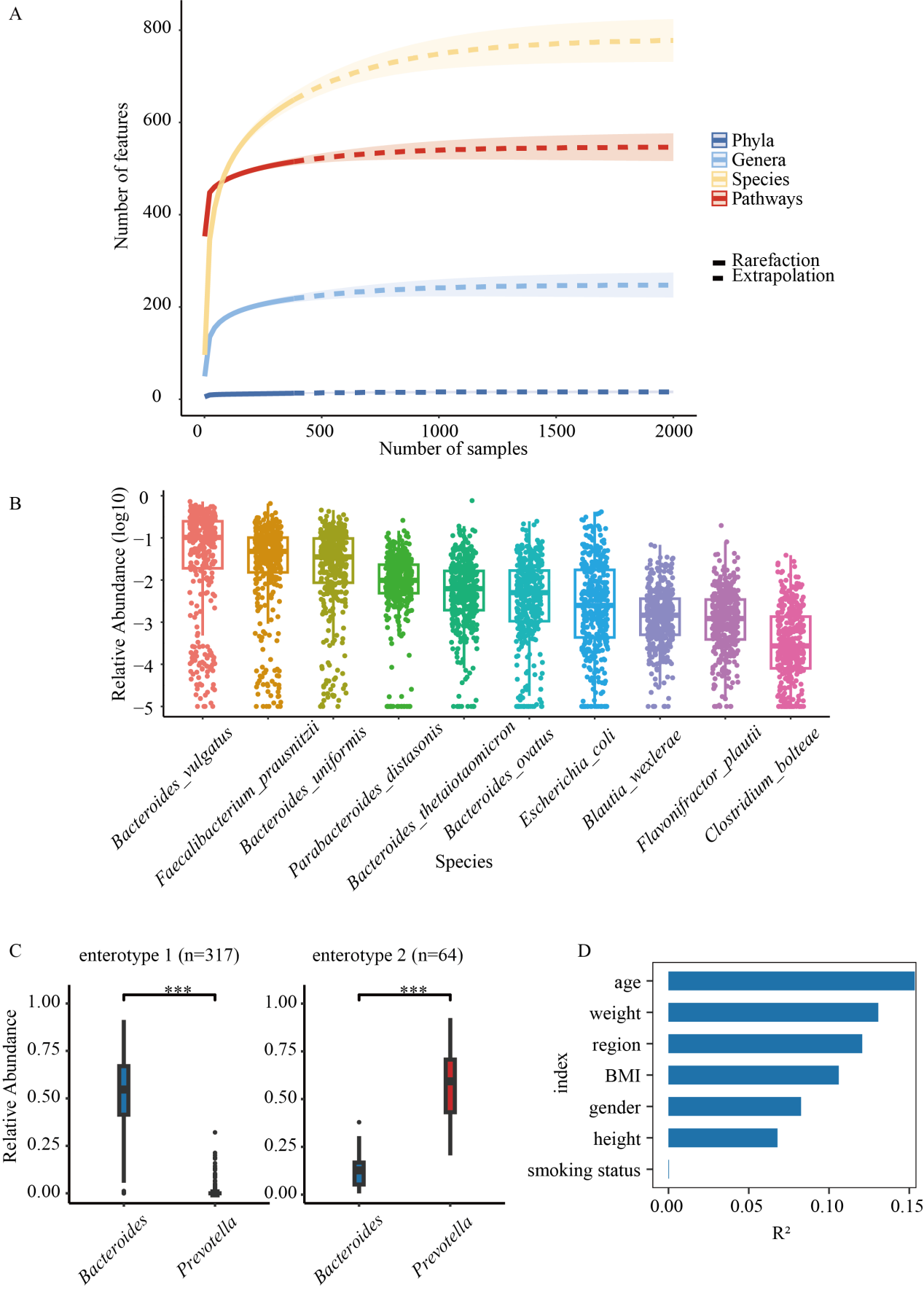


**Figure S2.** **Taxonomic composition of Hubei participants.** (A) Rarefaction and extrapolation sampling curve for microbiota features. (B) The relative abundances (log10) of the core species. (C) The boxplots show the top contributors at the genus level of each enterotype. (D) The effect sizes of personal characteristics associated with genera variations were calculated with envfit (vegan), all characteristics with FDR < 0.05.


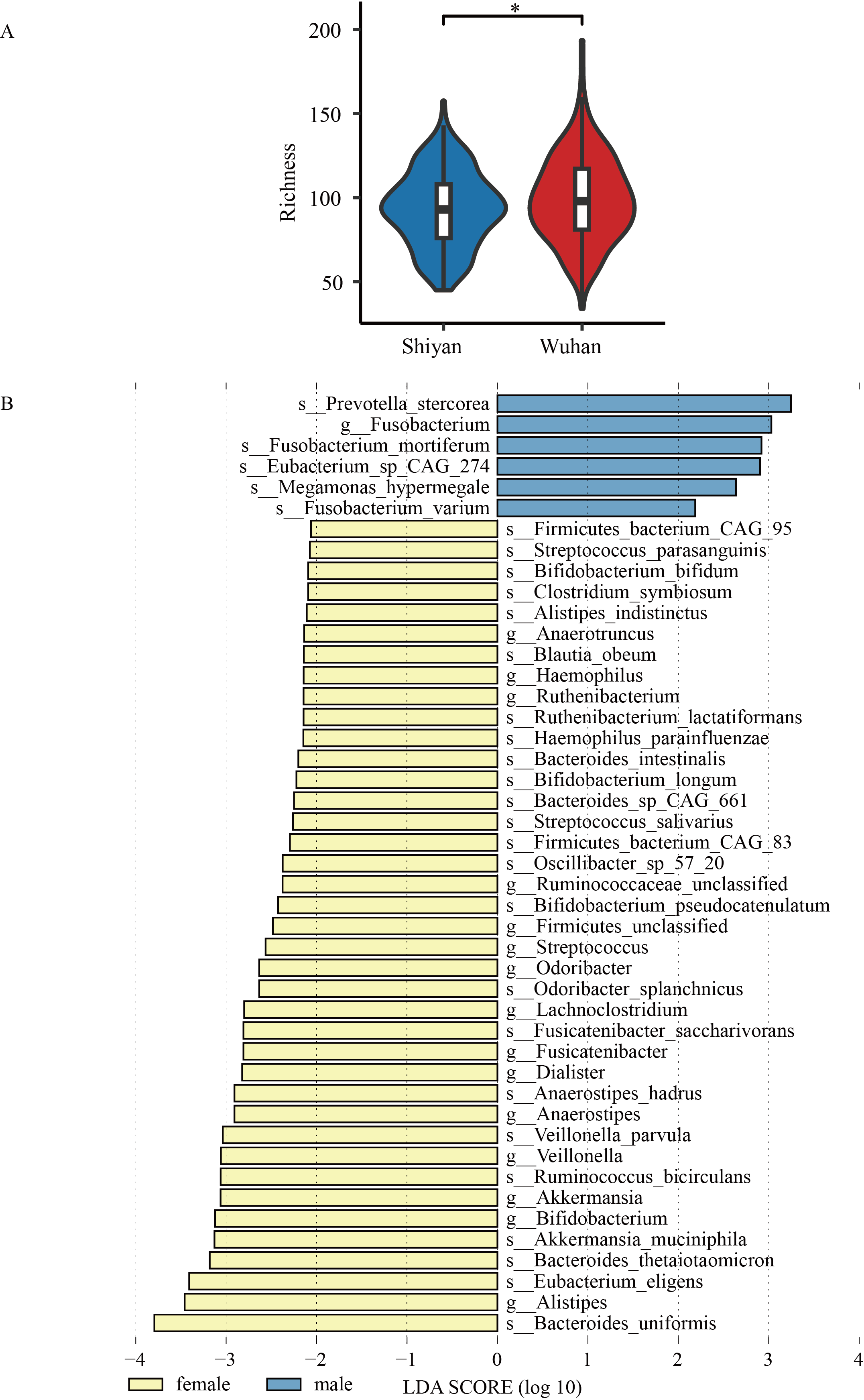


**Figure S3. Richness diversity between regions and microbiota with statistics difference between sexes.** (A) Richness diversity calculated on species-level taxonomic classifications for each sample. (B) Microbiota with statistics difference between sexes.


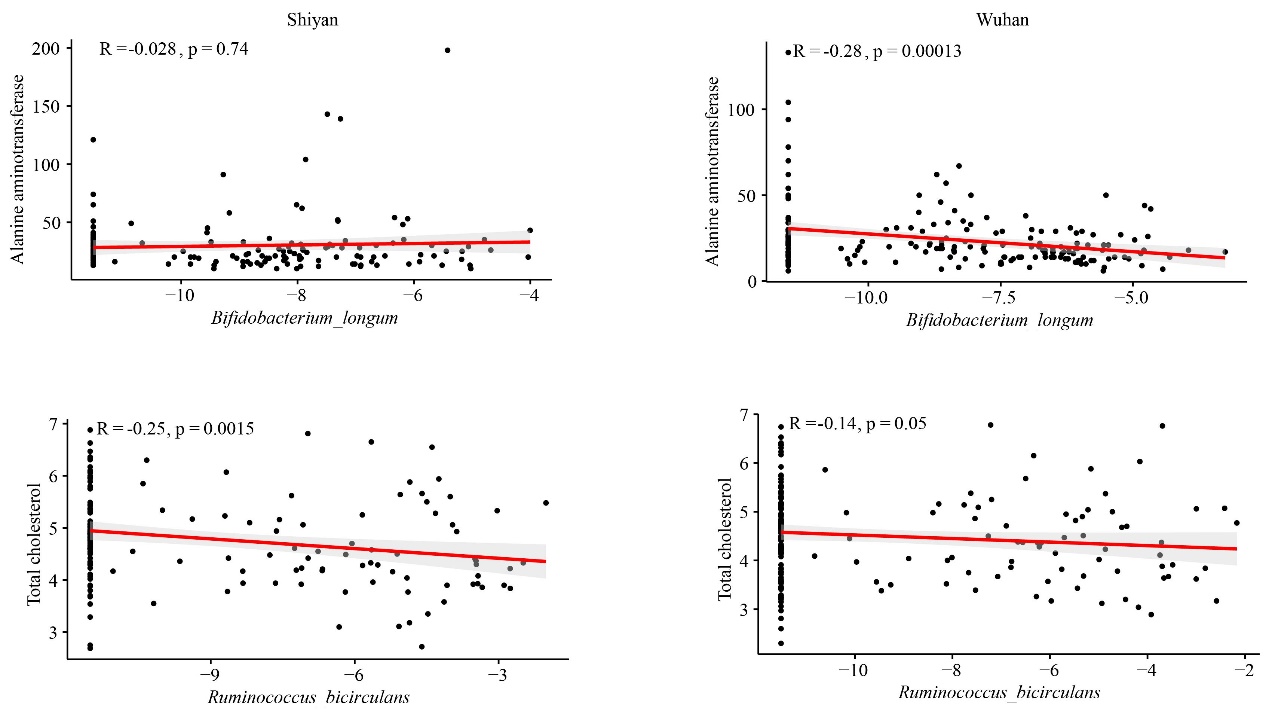


**Figure S4. Correlations between the relative abundance (log) of species and clinical measurements.**


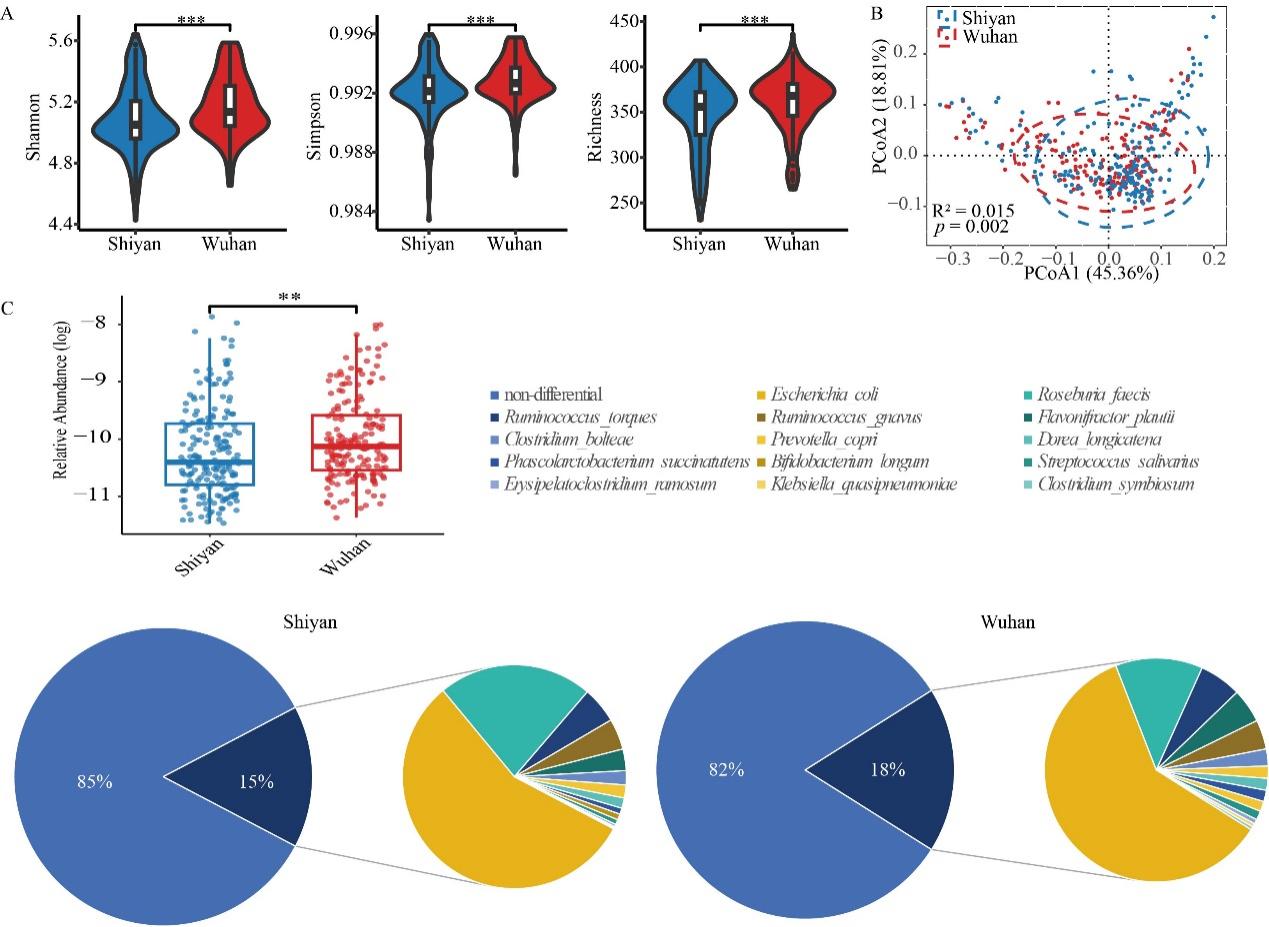


**Figure S5. Function diversity and species difference between Shiyan and Wuhan.** (A) Alpha-diversities of function between Wuhan and Shiyan (*p* value was calculated using a wilcoxon test; ****p* < .001). (B) PcoA of pairwise bray–curtis distance showed the separated function between two populations (Adnois R^2^ = 0.015, *p* < 0.01). (C) Distribution of bacteria involved in PWY-7111 (pyruvate fermentation to isobutanol that only enriched in Wuhan) in two cities.


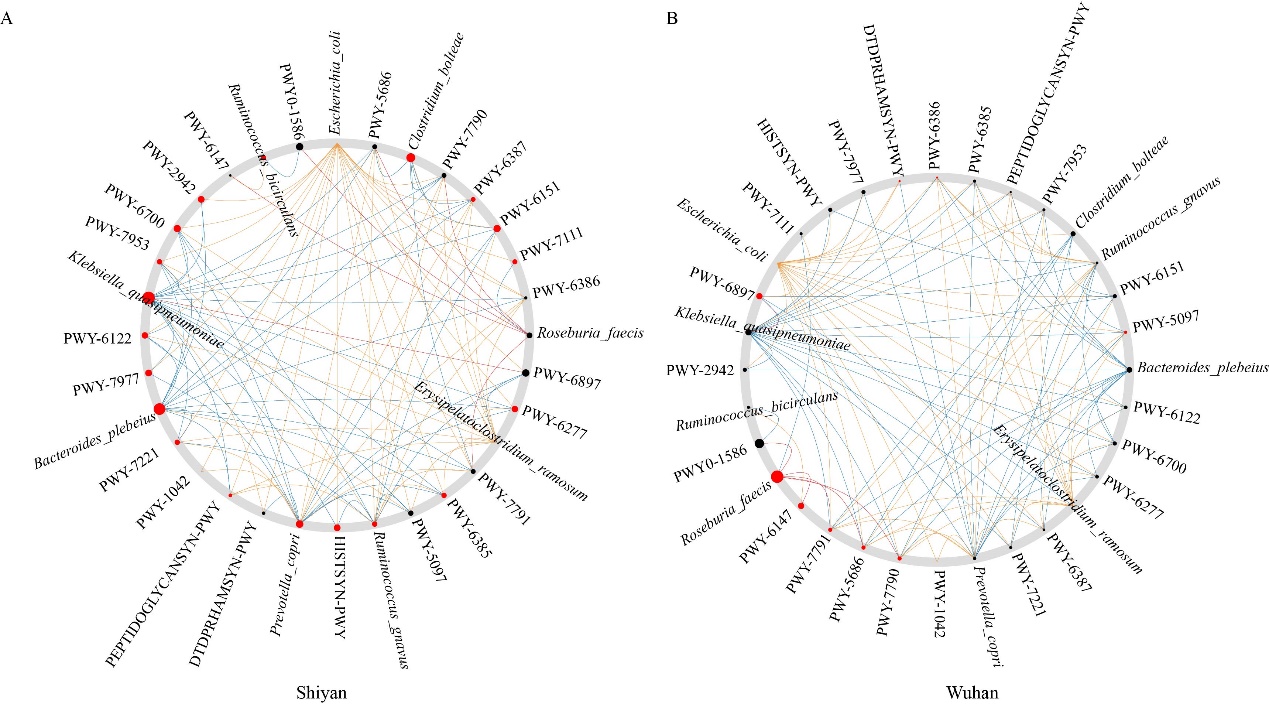


**Figure S6. The common sub-network between the Wuhan and Shiyan.** (A) Wuhan as control. (B) Shiyan as control. Node sizes were proportional to their scaled neighbor shift (NESH) score, and a node was marked as red if its betweenness increased from control to case. Significant “drivers” were nodes that were large and red. Red edges, only in Wuhan; blue edges, only in Shiyan; orange edges, present in both.


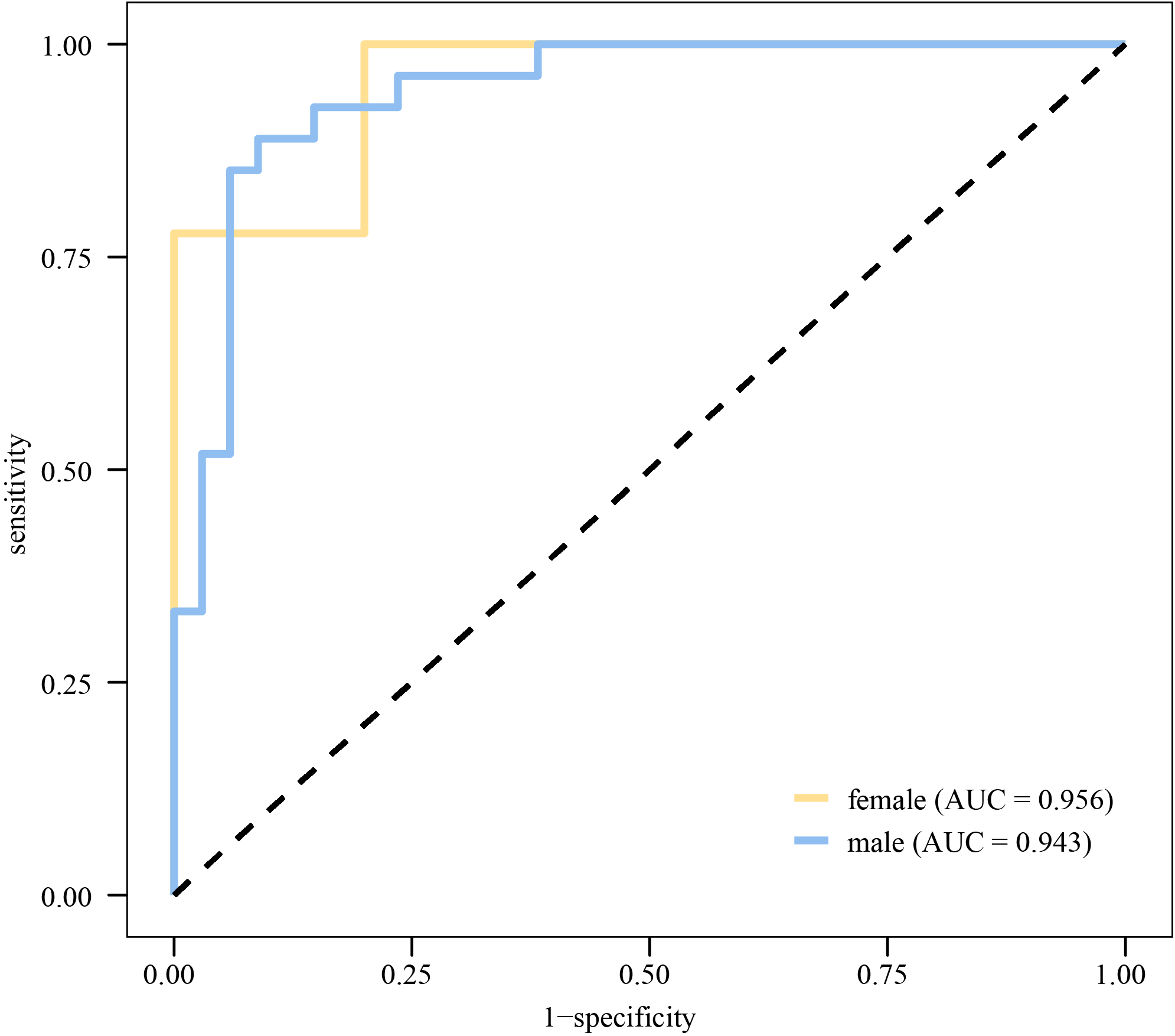


**Figure S7. The AUC of the optimal model under the sex subgroup.**
